# Amyloid fibril composition within hereditary Val30Met (*p*. *Val50Met*) transthyretin amyloidosis families

**DOI:** 10.1371/journal.pone.0211983

**Published:** 2019-02-27

**Authors:** Ole Bernt Suhr, Jonas Wixner, Intissar Anan, Hans-Erik Lundgren, Priyantha Wijayatunga, Per Westermark, Elisabet Ihse

**Affiliations:** 1 Department of Public Health and Clinical Medicine, Umeå University, Umeå, Sweden; 2 Department of Statistics, Umeå University, Umeå, Sweden; 3 Department of Immunology, Genetics and Pathology, Uppsala University, Uppsala, Sweden; Medizinische Fakultat der RWTH Aachen, GERMANY

## Abstract

**Background:**

The amyloid fibril in hereditary transthyretin (TTR) Val30Met (pVal50Met) amyloid (ATTR Val30Met) amyloidosis is composed of either a mixture of full-length and TTR fragments (Type A) or of only full-length TTR (Type B). The type of amyloid fibril exerts an impact on the phenotype of the disease, and on the outcome of diagnostic procedures and therapy. The aim of the present study was to investigate if the type of amyloid fibril remains the same within ATTR Val30Met amyloidosis families.

**Methods:**

Fifteen families were identified in whom at least two first-degree relatives had their amyloid fibril composition determined. The type of ATTR was determined by Western blot in all but two patients. For these two patients a positive 99mTc-3,3-diphosphono-1,2-propanodicarboxylic acid scintigraphy indicated ATTR Type A.

**Results:**

In 14 of the 15 families, the same amyloid fibril composition was noted irrespective of differences in age at onset. In the one family, different ATTR fibril types was found in two brothers with similar ages at onset.

**Conclusions:**

Family predisposition appears to have an impact on amyloid fibril composition in members of the family irrespective of their age at onset of disease, but if genetically determined, the gene/genes are likely to be situated at another location than the TTR gene in the genome.

## Introduction

Hereditary transthyretin amyloid (ATTRv) amyloidosis is a fatal autosomal dominant inherited systemic amyloidosis. The mechanism leading to amyloid formation has not been fully elucidated, and it appears that different pathways are operating [1]. The prevailing theory suggests that the majority of transthyretin (TTR) mutations lead to instability of the TTR tetramer; this further leads to dissociation of the tetramer into monomers that after misfolding subsequently reassemble into TTR amyloid (ATTR) fibrils [2]. However, ATTR rarely consists of only full-length TTR since C-terminal fragments, cleaved mainly at position 46, 49 or 52, are also often found in the deposits. In fact, ATTR fibrils consisting entirely of uncleaved TTR (Type B) so far have been found solely in patients with the TTR Val30Met *(p*. *Val50Met*), Phe64Leu (*p*. *Phe84Leu*), Glu74Asp (*p*. *Glu94Asp*), and Tyr114Cys *(p*. *Tyr134* Cys) mutations; furthermore, Type B fibrils mostly occur in a subgroup of patients that are predominantly characterised by early onset of their disease [3–6]. In addition, wild type TTR is also amyloidogenic, and wild type ATTR always consists of a mixture of cleaved and full-length TTR (Type A) [7, 8]. So far, the same amyloid fibril composition type has been found within an individual patient irrespective of the organ examined, e.g., the amyloid fibril composition in the heart is the same as in the subcutaneous fat [4].

The amyloid fibril composition has important clinical implications, both regarding diagnostic tests, phenotype of the disease, and on response to treatment since early onset ATTR Val30Met amyloidosis patients, who predominantly carry type B fibrils, display superior survival after liver transplantation and after Tafamidis (a TTR stabiliser) treatment [1, 9–11]. In addition, after liver transplantation, a slower increase of wild type TTR in type B patients’ ATTR deposits were found compared with that for type A [12]. Marked difference in phenotype have also been observed, based on the Congophilic properties of ATTR deposits that reflect the amyloid fibril composition, where type A fibrils display low affinity for Congo red staining in contrast to a high affinity for type B fibrils. Thus, for patients with an early onset originating from endemic areas, a higher amount of variant TTR was found in their ATTR deposits that displayed a high affinity for Congo red staining [13]. Interestingly, differences have also been noted for nerve damage, where the classical small fibre peripheral neuropathy was related to elongation of the ATTR fibrils, i.e., a finding noted in patients with type B amyloid deposits [7, 14].

The mechanism behind the formation of two fibril types is virtually unknown. A mechano-enzymatic cleavage process has been suggested [15] where mechanical force on the circulating TTR in conjunction with its passage through the heart leads to a dislocation of the TTR’s D-loop, which is then exposed to enzymatic cleavage by plasmin [16]. After this the tetramer is broken up into monomers facilitating formation of amyloid fibrils consisting of C-terminal TTR fragments and full-length TTR, i.e., Type A fibrils. However, Type A fibrils have also been found in material of the corpus vitreous obtained from vitrectomised ATTR Val30Met amyloidosis patients’ eyes, and in the spinal cord of diseased ATTRVal30Met patients [6, 17]. Since a mechano-enzymatic cleavage of TTR is unlikely to occur in the eye or central nervous system alternative mechanisms must be operating.

The aim of the present study was to investigate the relationship between family membership and amyloid fibril composition in ATTR Val30Met amyloidosis patients, in an attempt to better understand the underlying cause of the two fibril conformation types. The hypothesis was that amyloid fibril composition is inherited within families, i.e., determined by genetic factors. Our results support the hypothesis.

## Material and methods

To evaluate the ATTR fibril composition within ATTR Val30Met families, we conducted a retrospective study on ATTR Val30Met amyloidosis families at our centre, which is a tertiary reference centre for ATTR amyloidosis.

During the last 10 years, we have performed fat pad biopsies with typing of amyloid deposits, and if ATTR is found we determined the type of ATTR fibril, i.e., Type A or Type B. To explore the families and their amyloid fibril types, we scrutinised our patient material at the Amyloid Centre at Umeå University Hospital for ATTR Val30Met families in which two or more first degree relatives had had their amyloid fibril composition determined. The diagnosis in all patients was verified by positive gene sequencing or PCR examination for the TTR Val30Met mutation. Amyloid deposition was detected in an abdominal fat pad biopsy by Congo red staining and examination under polarised light with crossed polars. The type of ATTR fibril was determined by Western blot utilising a rabbit antiserum against an *in vitro* expressed C-terminal fragment of TTR (TTR50-127), diluted 1:5000 that recognises both full-length and C-terminal TTR fragments as previously described [4]. In two patients, the fibril type was settled by 99mTc-3,3-diphosphono-1,2-propanodicarboxylic acid (DPD) scintigraphy [18]. Prism 7 statistical software was used for statistical analysis. Differences between groups were analysed by the Mann-Whitney test. To analyse the probability of finding the same fibril type within the families, empirical probabilities were determined and bootstrap confidence intervals were calculated from 10,000 resamples with replacements of same sample size from the original sample of observations.

The study was approved by the Regional Ethics Committee at Umeå University. Dnr 2017-447-31M.

## Results

**Fig 1:**
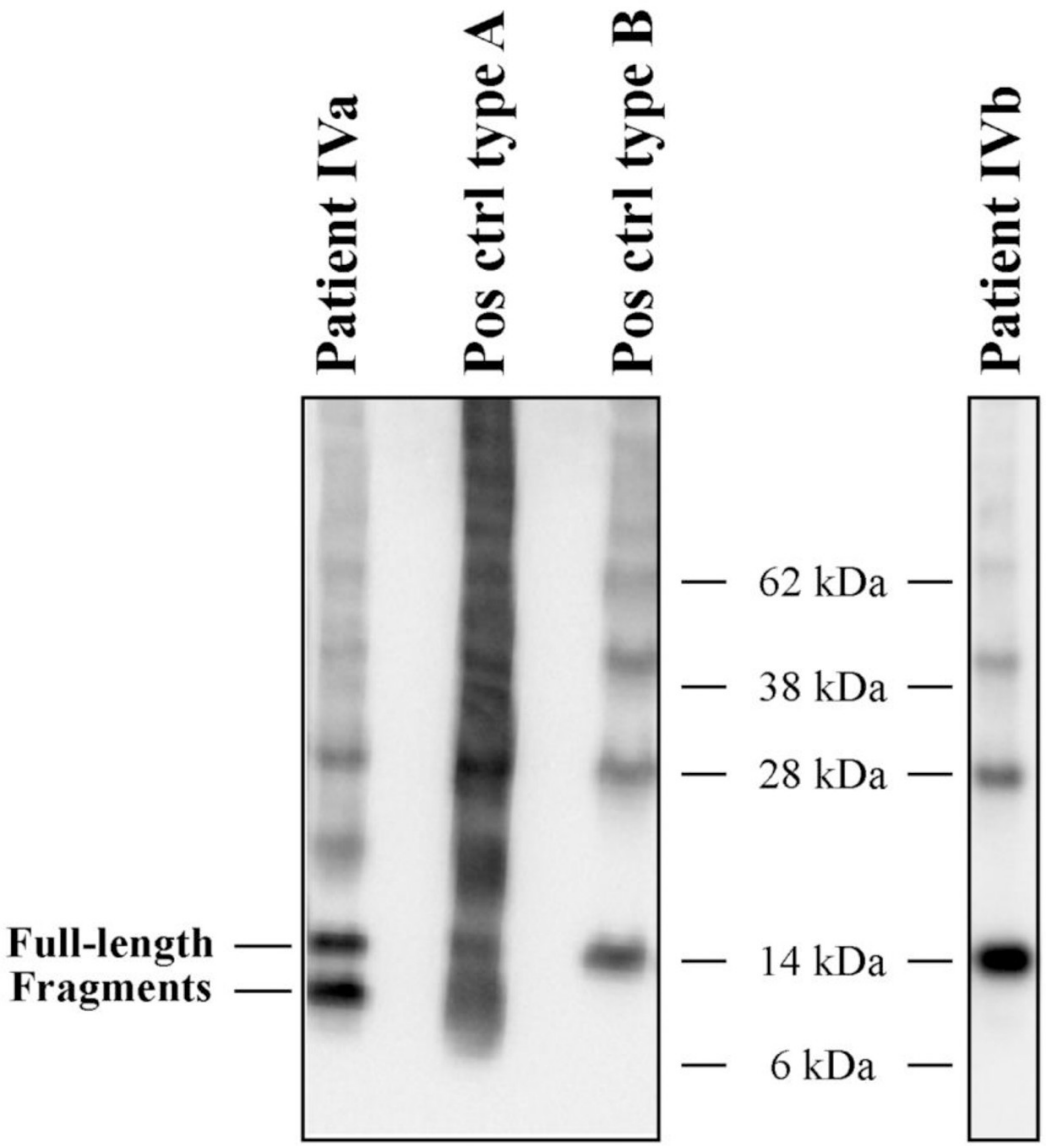
The two fibril types shown by western blotting analysis. Western blots subjected to an antiserum directed against the C-terminal part of transthyretin (amino acids 50–127) are shown for patient IVA and IVB, as well as positive controls for both fibril types. Patient IVA is seen to have a clear band below the band corresponding to the full-length monomeric transthyretin, while patient IVB does not.

Fifteen families in which at least two first degree family members had had their amyloid fibril composition determined were identified. The total number of patients was 31. The type of ATTR was settled by Western blot in all but two patients. For these two patients, for whom the amyloid content in the fat pad biopsy was insufficient for typing, a positive DPD-scintigraphy suggested ATTR Type A, as it has previously been shown that patients with Type B fibrils have negative DPD-scintigraphies [18]. The families and their amyloid fibril composition type together with age at onset and outcome of DPD-scintigraphy are summarised in Table 1. In 14 of the 15 families, the same amyloid fibril composition was noted in the family members, while in one family, two brothers, both with an onset of their disease at the age of 67 years, displayed different fibril types (Fig 1) (family IV). The empirical probability of observing the same fibril type within a family was 0.93 with a bootstrap 95% confidence interval (CI) 0.8–1.0. Corresponding values calculated from those families with confirmation of their fibril type by Western blot were 0.93 with a bootstrap CI of 0.7857–1.

**Table 1:** Amyloid fibril composition within the families.

*Family*	*Family members*	*Age at onset of disease*	*Index Patient’s trait from*	*Fibril type by DPD-scintigraphy*^a^	*Fibril type by Western blot*	*Fibril type by western blot/DPD-scintigraphy*
Ia	index male	48	mother	ND^b^	B^c^	B
Ib	son	44		B	B	B
IIa	index female	44	unknown	B	B	B
IIb	son	44	B	B	B	B
IIIa	index female	67	unknown	A^d^	A	A
IIIb	brother	54		A	NA^e^	A
IIIc	son to IIIb	46		A	A	A
IVa	index male	67	father	A	A	A
IVb	brother	67		B	B	B
Va	index male	67	father	A	A	A
Vb	brother	71		A	A	A
VI	index female	62	father	ND	B	B
VIb	daughter	49		ND	B	B
VIIa	index female	60	unknown^f^	B	B	B
VIIb	brother	62		ND	B	B
VIIIa	index male	69	mother	ND	B	B
VIIIb	son	44		ND	B	B
IXa	index male	43	father	B	B	B
IXb	brother	38		ND	B	B
Xa	index male	39	mother	B	B	B
Xb	brother	38		B	B	B
XIa	index male	63	mother	ND	B	B
XIb	son	48		B	B	B
XIIa	index male	32	mother	ND	B	B
XIIb	brother	28		ND	B	B
XIIIa	index male	72	father	A	NA	A
XIIIb	brother	64		A	A	A
XIVa	index male	36	father	B	B	B
XIVb	brother	29		B	B	B
XVa	index male	71	Father	A	A	A
XVb	sister	65		A	A	A

^a^ DPD—scintigraphy = 99mTc-3,3-diphosphono-1,2-propanodicarboxylic acid-scintigraphy, where negative scintigraphy relates to Type B fibrils, whereas positive scintigraphy relates to type A fibrils

^b^ not done

^c^ Type B amyloid fibrils consisting of full-length transthyretin only.

^d^ Type A amyloid fibril consisting of a mixture of full-length and truncated transthyretin

^e^ Amyloid fibril composition could not be determined by Western blot

^f^ both parents deceased without symptoms of ATTR amyloidosis, but the disease was present in both parents’ families.

Six families contained a parent and a child, and for three of these families (VI, VIII and XI), there was a disparity in the age of onset of more than 10 years (13, 25 and 15 years, respectively).

**Fig 2:**
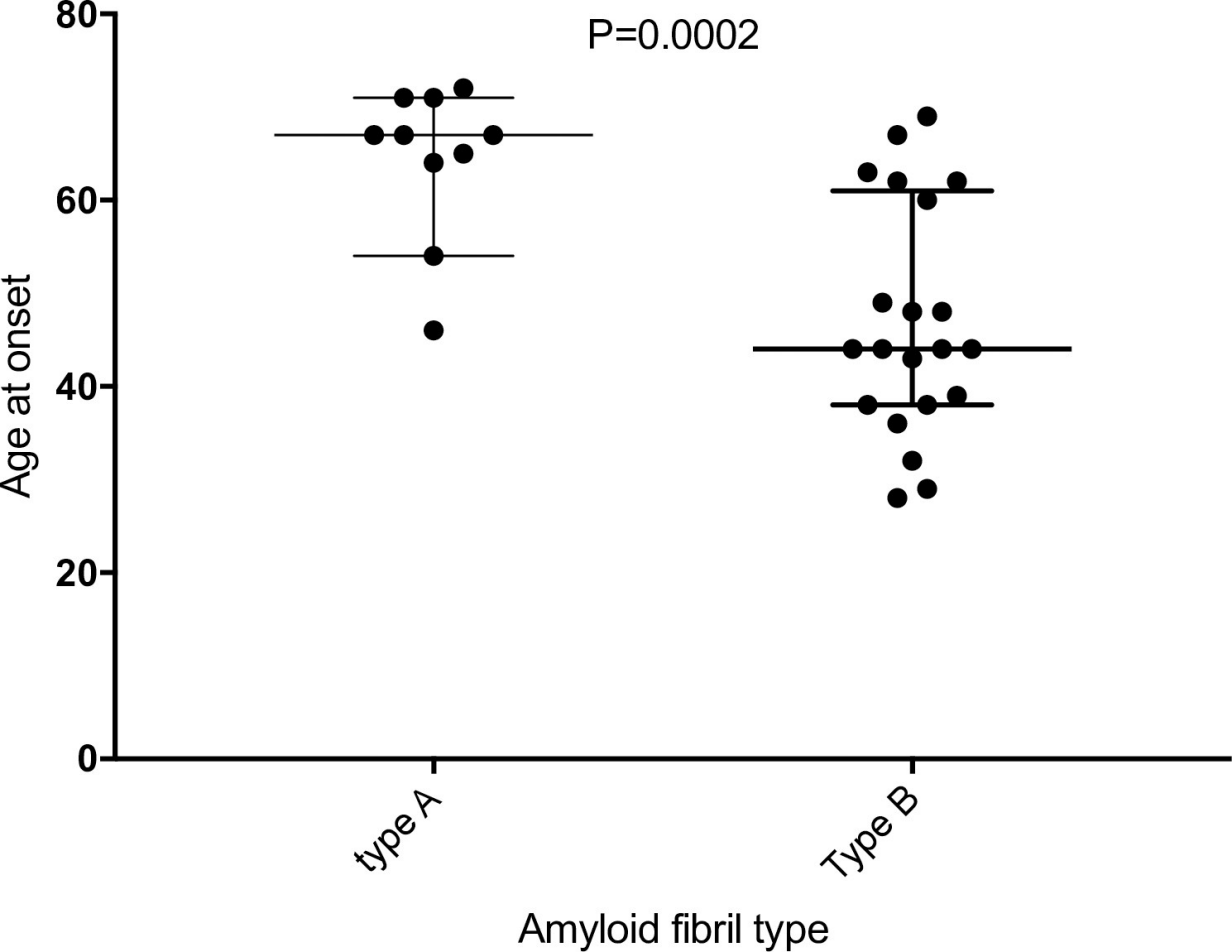
Scatterplot of amyloid fibril type and age at onset. Type A fibrils contain transthyretin fragments. Type B fibrils contain only full-length transthyretin. Bars represent median and 95% confidence interval.

Expectedly, patients with Type A ATTR fibrils were significantly older at disease onset than those with Type B fibrils (median 67 and 44 years respectively; P < 0.0002; Fig 2). However, in line with what was shown previously, there was an overlap between the fibril types and age of onset such that either fibril type can be found in patients with an age of onset between approximately 45–70 years.

## Discussion

The most important finding was the consistency of amyloid fibril composition within all but one of the families. This substantial consistency of amyloid fibril composition type within the families, often in spite of a considerable difference in age at onset (up to 25 years) suggests that genetic factors play a role.

Generally, late onset patients display Type A, and early onset patients display Type B fibrils. However, it should be noted that there is not a pure correlation between fibril type and the traditional grouping of patients as early (age of onset below 50 years) and late (age of onset above 50 years) onset cases. While the majority of early onset patients will have type B fibrils, late onset patients with an age of onset below 70 years of age may also have Type B fibrils.

Domino liver transplant recipients display a similar consistency of amyloid fibril composition after transferring a mutant ATTR producing liver to a patient with end-stage liver disease. So far, all recipients of an ATTR Val30Met liver from a patient with Type B fibrils have displayed Type B fibrils irrespective of their age when they received the liver [19]. This could indicate that the determination of ATTR fibril type is regulated within the liver after synthesis of TTR, and is not dependent on extra-hepatic factors. However, we have not yet had the opportunity to examine patients receiving livers from patients displaying Type A fibrils.

The typing of amyloid deposits can be challenging. In the presented series of patients, amyloid fibril composition could not be determined from performing western blotting on fat biopsies in two patients. One patient underwent two abdominal fat pad biopsies after an initial positive heart biopsy, and even though amyloid was detected in the second fat pad biopsy, the amount was too small for Western blot analysis. In the other patient, the amount of amyloid in the fat pad biopsy was also too small for fibril typing. However, as previously reported, patients with Type A amyloid fibril generally have positive DPD scintigraphies, even in patients with normal heart dimensions, while patients with type B fibrils have negative scintigraphies [18]. Therefore, it appears reasonable to accept that the patients for whom the amyloid fibril composition could not be determined from fat pad biopsies had Type A fibrils based on their positive scintigraphic findings. In addition, the amount of amyloid in fat pad biopsies are generally lower in patients with Type A fibrils and displays a lower affinity for Congo red staining [7, 20].

Another limitation of the study is the small number of examined families, which is mainly due to the relatively low number of patients diagnosed yearly in combination with a relatively short period of time in which we routinely have determined the amyloid fibril composition type. In addition, we have only included first-degree relatives in the present study.

## Conclusion

The consistency of amyloid fibril composition type within 14 of 15 investigated families suggests that genetic/epigenetic factors exert an impact on amyloid fibril composition. However, the finding of different types of fibrils in a pair of brothers, both with the same age at onset suggests that the gene/genes regulating TTR cleavage is situated at another location than the TTR gene on the genome.
